# Antibody Titres to Strangvac^®^ Antigens Correlate with Protection and Duration of Immunity Against Experimental Infection with *Streptococcus equi* Subspecies *equi*

**DOI:** 10.3390/vaccines14060533

**Published:** 2026-06-16

**Authors:** Romain Paillot, Francesco Righetti, Carl Robinson, Lars Frykberg, Margareta Flock, Olof Zachrisson, Bengt Guss, Jan-Ingmar Flock, Andrew S. Waller

**Affiliations:** 1 Intervacc AB, 129 44 Stockholm, Swedenandrew.waller@intervacc.com (A.S.W.); 2 Department of Microbiology, Tumor and Cell Biology, Karolinska Institutet, 171 77 Stockholm, Sweden; francesco.righetti@ki.se (F.R.); jan-ingmar.flock@ki.se (J.-I.F.); 3 Department of Bacteriology, Animal Health Trust, Lanwades Park, Kentford, Newmarket CB8 7UU, UK; 4 Department of Animal Biosciences, Swedish University of Agricultural Sciences, 750 07 Uppsala, Sweden; lars.frykberg@slu.se (L.F.); bengt.guss@slu.se (B.G.)

**Keywords:** strangles, *Streptococcus equi*, vaccination, correlate of protection, IdeE, antibody response

## Abstract

**Background/Objectives:** Strangles, caused by *Streptococcus equi* subspecies *equi* (*S. equi*), remains a common and severe equine infectious disease. Strangvac^®^, a recombinant fusion protein vaccine licenced in Europe, contains the antigens (Ag) CCE, Eq85, IdeE and a saponin adjuvant. Although its efficacy is high (94% in clinical trials and 100% in some natural outbreaks), immune correlates of protection have not been defined. This study determined the antibody (Ab) thresholds predictive of protection against clinical disease following high-dose experimental *S. equi* infection and the expected levels of protection at 6 and 12 months after V2. **Methods:** This study was a retrospective analysis of six independent double-blinded placebo-controlled experimental infection studies involving 129 ponies (80 vaccinated controls and 49 placebo controls) and a serology study (12 vaccinated ponies). Ponies received two to five vaccine doses before being experimentally challenged with *S. equi* strain *Se*4047. Ponies in the serology study were not experimentally infected. The onset of pyrexia (≥39 °C for at least 2 of 3 consecutive days, OOT) was used as a disease marker. Serology to IdeE, Eq85 and CCE was analysed with standardised clinical outcomes to define protective thresholds through correlation and Receiver Operating Characteristic (ROC) analyses. The predicted level of protection up to one year after V2 was then calculated (duration of immunity: DOI). **Results:** A protection threshold of ≥10 days to OOT, derived from the control distribution, was used for ROC modelling. Predictive performance (e.g., accuracy, precision, specificity) was calculated for individual and combined Ab thresholds. All controls developed pyrexia (median 6 days, IQR 5–7), with 46 out of 49 (93.9%) within 9 days of the challenge. Vaccinated ponies showed significantly delayed or absent OOT compared with controls (*p* < 0.0001), with 37 vaccinated ponies (46.25%) reaching the end of the studies without developing pyrexia. The Ab titre to all antigens was significantly associated with the level of protection (*p* < 0.0001). ROC analyses demonstrated high discriminative power (AUC 0.86–0.88). Optimal Ab titre boundaries yielded high precision (≥80%) for all Ags (IdeE: 3.5–4.3; Eq85: 2.65–3.7 and CCE: 2.66–3.2). Both precision and accuracy remained above 80% for levels of IdeE and Eq85 Ab titres superior or equal to those measured up to one year after V2, with an estimated level of protection of 78.9% to 81.2% in vaccinated animals. **Conclusions:** Ab titres to all three Ags represent robust correlates of protection against pyrexia following high-dose experimental *S. equi* challenge in Strangvac^®^-vaccinated ponies. Ab titres measured up to one year after V2 were estimated to continue to provide significant protection in vaccinated animals. These findings support the observed levels of protection conferred by Strangvac^®^ against natural infection with *S. equi*.

## 1. Introduction

Strangles, caused by *Streptococcus equi* subspecies *equi* (*S. equi*), remains one of the most frequently diagnosed infectious diseases of horses [1,2,3]. One of the first clinical signs of strangles is an abnormal elevation of body temperature, followed by swelling and abscess formation in the lymph nodes (LN) of the head and neck, which characterise the disease. Mucopurulent nasal discharges are usually the consequence of the rupture of an abscessed retropharyngeal LN. The acute form of strangles can last several weeks and can lead to life-threatening complications, such as purpura haemorrhagica (immune-mediated vasculitis) and disseminated infection (metastatic abscess formation, also called bastard strangles). Mortality may occur in up to 10% of cases depending on the health and immune status of the affected horses [4,5,6,7,8]. *S. equi* persists in the guttural pouches of approximately 10% of recovered horses, which may remain carriers of *S. equi* for several years after the original outbreak [9,10,11].

The welfare and economic impact of strangles is significant. An outbreak can last several months and usually requires intense veterinary intervention for resolution and for any persistently infected carriers to be identified and confirmed to be clear of bacterial persistence. The overall financial cost ranges from €170 to €111,874 per outbreak, depending on the numbers of horses affected and whether complications occur [12,13,14]. Although it has too often been neglected in the past, the adverse psychological impact on horse owners, yard owners and veterinarians is also a very important consideration. As with most infectious diseases, hygiene and biosecurity measures can mitigate the risk of *S. equi* introduction, infection and the development of outbreaks, but vaccination has become the primary and most effective method of prevention [12].

**Figure 1:**
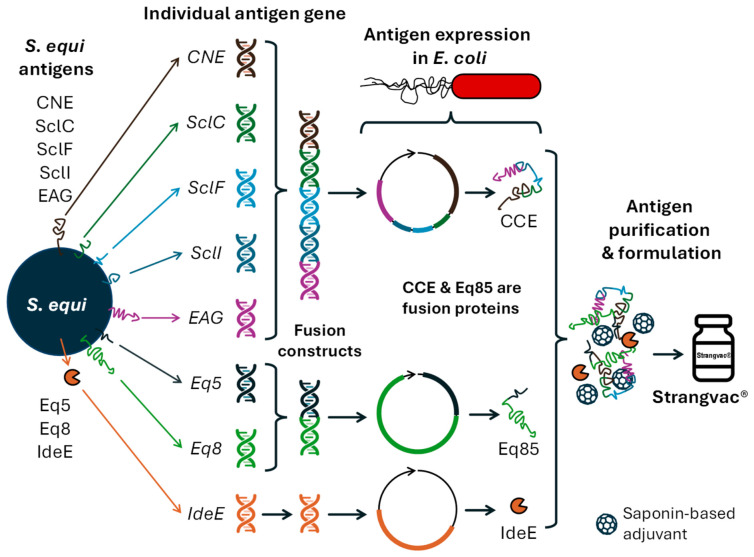
Summary of the fusion proteins contained in Strangvac^®^, reprinted with permission from Ref. [13], 2026, @ Romain Paillot.

Strangvac^®^ is a recombinant fusion protein vaccine which protected 94% of horses from an experimental high-dose challenge during a clinical trial [15]. The vaccination of horses in field conditions is significantly associated with protection against natural exposure to *S. equi* [16,17,18]. Strangvac^®^ contains fragments of the *S. equi* cell surface proteins CNE, SclC, SclF, SclI, and EAG that are fused together to form the vaccine antigen (Ag) CCE, fragments of the cell surface proteins Eq8 and Eq5 that are combined as vaccine antigen Eq85, the secreted protein IdeE, and a saponin-based adjuvant (Figure 1).

The biological function of the sortase-processed surface proteins SclC, SclF, SclI, Eq8 and Eq5 remains to be defined, but they have been shown to contribute to the protection provided by vaccination with Strangvac^®^ [15]. CNE is a collagen-binding surface protein [19]. EAG is a protein G-related cell surface protein that binds to alpha 2 macroglobulin, albumin and IgG [20]. Antisera specific to EAG increased opsonophagocytic activity in vitro [21]. One of the vaccine antigens, IdeE, is an endopeptidase-like virulence factor used by *S. equi* to degrade immunoglobulins [22], which probably allows it to evade immune mechanisms such as opsonization (facilitation of recognition by immune cells), phagocytosis and/or activation of the classical complement pathway (recognition of the antibody-pathogenic immune complex by the C1q molecule), thus promoting its survival. Righetti et al. (2025) demonstrated that the antibody (Ab) response induced by Strangvac^®^ neutralised IdeE endopeptidase activity in vitro [23]. The neutralisation of this virulence mechanism is likely to play an important role in the protection induced by Strangvac^®^. The nature and known function of the Ags incorporated in Strangvac^®^ are summarised in Table 1.

A correlate of protection is a measurable immunological marker that is statistically associated with protection against infection, clinical signs of disease and other clinically relevant outcomes, and/or the dissemination and transmission of pathogens. Correlates of protection allow the assessment of protective immunity following vaccination, and enable prediction of expected outcomes in the event of exposure to the pathogen of interest [26]. Only a few correlates of protection have been defined for equine infectious diseases. For equine influenza (EI), equine influenza virus (EIV)-specific single radial haemolysis (SRH) Ab titres have been established as correlates of protection. Defined SRH Ab thresholds are associated with a significant reduction in the clinical signs of disease or virus shedding (≥85 mm^2^ and ≥154 mm^2^, respectively), provided that the infecting EIV strain is antigenically similar to the vaccine strains [27,28]. This correlate is valuable not only for evaluating expected protection in individual horses but also for determining herd immunity [29] and for comparing the immunogenicity of different EI vaccines [30]. The EIV-specific Ab titre can also be used as a surrogate of protection (used as a replacement of clinical endpoint studies) in the context of updating EIV vaccine strains in the EU. This approach simplifies the registration process for partially updated EI vaccines by inferring protection against a substituted EIV vaccine strain when equivalence of Ab response is demonstrated, compared with the clinically proven serological response to other EIV strains in the same vaccine [31]. Similarly, a level of tetanus toxin binding Ab greater than 0.01 IU/mL is associated with protection in horses [32] and Ab titres are also used to assess vaccination schedules [33]. Virus-neutralising (VN) Ab titres at the time of challenge have been correlated with clinical protection against equine arteritis virus (EAV) infection and with a reduction in virus shedding [34]. Nevertheless, additional immune mechanisms are likely to also contribute to protection against EAV [35]. Similarly, a VN Ab titre of 32 is considered a presumptive protective titre in horses against Hendra virus infection [36,37].

While the humoral immune response after two doses of Strangvac^®^, including the functional neutralisation of IdeE endopeptidase activity [23], is well documented, no immune correlate of protection has yet been defined. This retrospective study aimed (1) to determine the Ab thresholds associated with significant levels of protection against clinical disease induced by *S. equi* infection following experimental high-dose challenge and (2) to model the expected duration of immunity against *S. equi* based on Ab levels measured after two administrations of Strangvac^®^.

## 2. Materials and Methods

### 2.1. Study Design

The serology and clinical data that were retrospectively analysed in this study were collected from seven independent clinical trials (Study #1–#7). All studies are summarised in Table 2. Five of the studies (Study #1–#4 and #7) were previously reported in [15,38]. Two studies (Studies #5 and #6) remain unpublished, but clinical and serological data were made available for the current report. A study outline for these two unpublished clinical trials is presented in Supplementary File S1.

Depending on the study, vaccinated ponies were administered between two and five intramuscular doses of Strangvac^®^. Controls received the adjuvant only. All ponies (≤2 years of age) were experimentally infected with *S. equi* (*Se*4047 strain, 1.05–2.3 × 10^8^ CFU/pony) and clinical signs were monitored for three to four weeks, depending on the study.

Serology data were also obtained from a duration of immunity (DOI) study (Study #7) [15]. Ponies received 2 administrations of Strangvac^®^ 4 weeks apart and a third boost injection (V3) 91, 182 or 364 days after V2. These ponies were not experimentally infected with *S. equi*.

These experiments were conducted under the conditions of a Home Office Project License according to the Animal Scientific Procedures Act 1986 and following ethical review and approval by the Animal Health Trust’s Animal Welfare and Ethical Review Body (Research Project Proposal (RPP) 01_08; Approved in May 2008, reviewed and approved in December 2012). Further animal welfare information for these studies is included in Supplementary File S2.

### 2.2. Clinical Data

Clinical data were obtained from Study #1–#6, which included 80 vaccinated Welsh mountain ponies and 49 controls. The detail of the clinical examination scoring is presented in Supplementary Table S1. Rectal body temperature (measured in °C) was used as a non-subjective measurement of health and disease (Studies #1–#6; detailed in Supplementary Table S2). Temperatures ≥ 39.0 °C were considered abnormal. The onset of abnormal temperature (OOT) was defined as the first day with a rectal temperature ≥ 39.0 °C for at least 2 out of 3 consecutive days. For ponies that did not develop abnormal temperature, the last day of observation was considered for the purposes of analysis. Ten ponies reached the humane endpoint of the study (i.e., before the end of the study) without developing abnormal temperature. Thirty-seven reached the end of the study observation period without developing abnormal temperature (all in the vaccinated groups; right-censored for statistical analyses). Studies #1–#3 included a 21-day post-challenge observation period following experimental infection with *S. equi*. Studies #4 included 28 days of observation, Study #5 included 25 days, and Study #6 included 22 days. Despite these differences in monitoring duration that only affected vaccinated ponies, data were not truncated to the lowest study’s duration.

### 2.3. Quantification of the Antibody Response to Vaccination and Clinical Data

Serum IgG Ab titres to the vaccine Ags IdeE, Eq85 and CCE were measured at the time of the studies in 80 vaccinated Welsh mountain ponies and 40 controls by conventional iELISA performed as previously described [15]. Serum Ab titres measured at the time of experimental infection with *S. equi* were utilised in the current analysis (Studies #1–#6; detailed in Supplementary Table S2). Serology data from samples collected from 9 control ponies (Study #4 and Study #6) were not available. For the Ab response kinetics and DOI analysis, serology data were obtained from Studies #1–#3 and Study #7. Serum IgG Ab titres to each vaccine Ag were measured in vaccinated animals; the sampling time points per study are presented in Supplementary Figure S1.

### 2.4. Statistical Methods

The Shapiro–Wilk test was used to test for normality. For non-normally distributed data, the median and the interquartile range (IQR) are also reported. The Kruskal–Wallis H test was used to compare OOT between the different control groups, with a post hoc Dunn’s test and Bonferroni correction for pairwise comparisons. The Kaplan–Meier and log-rank test were used to compare the OOT from controls and vaccinated ponies. For multiple comparisons, the pairwise log-rank test with Benjamini–Hochberg (BH) False Discovery Rate correction was used. The one way ANOVA and Kruskal–Wallis H test were used to compare Ab titres between controls, vaccinated with OOT and vaccinated without OOT, with a post hoc Tukey HSD test and Dunn’s test, respectively, and Bonferroni correction for pairwise comparisons. The Mann–Whitney U test was used to compare Ab titres between protected and unprotected ponies. Fisher’s exact test (2 × 2 and 2 × 3) with Cramér’s V effect size interpretation was used for association analysis (i.e., vaccination status in relation to OOT) [39]. Significance was set at *p* < 0.05. Receiver Operating Characteristic (ROC) curve analyses were performed and the reported accuracies were calculated as follows (true positive + true negative)/(sum of all positives and negatives = 120).

For the ROC curve analyses, discriminative performance was assessed using the area under the ROC curve (AUC). Statistical significance was evaluated using a Monte Carlo-approximated permutation test based on 10,000 random permutations of the class labels, generating an empirical null distribution corresponding to chance-level discrimination. A Cox proportional hazards model was used for detecting associations between Ab titres and OOT because this test is designed to incorporate right-censored observations through partial likelihood estimation [40,41] and was associated with the AFT (Accelerated Failure Time) Log-Normal model. Significance was set at *p* < 0.05. Statistics Kingdom 2017, available from: http://www.statskingdom.com (first accessed on 15 January 2026) and R version 4.5.2 (Monte Carlo-approximated permutation test, Cox proportional hazards model and Kaplan–Meier with pairwise log-rank analysis).

## 3. Results

### 3.1. Onset of Abnormal Temperature (OOT)

**Figure 2:**
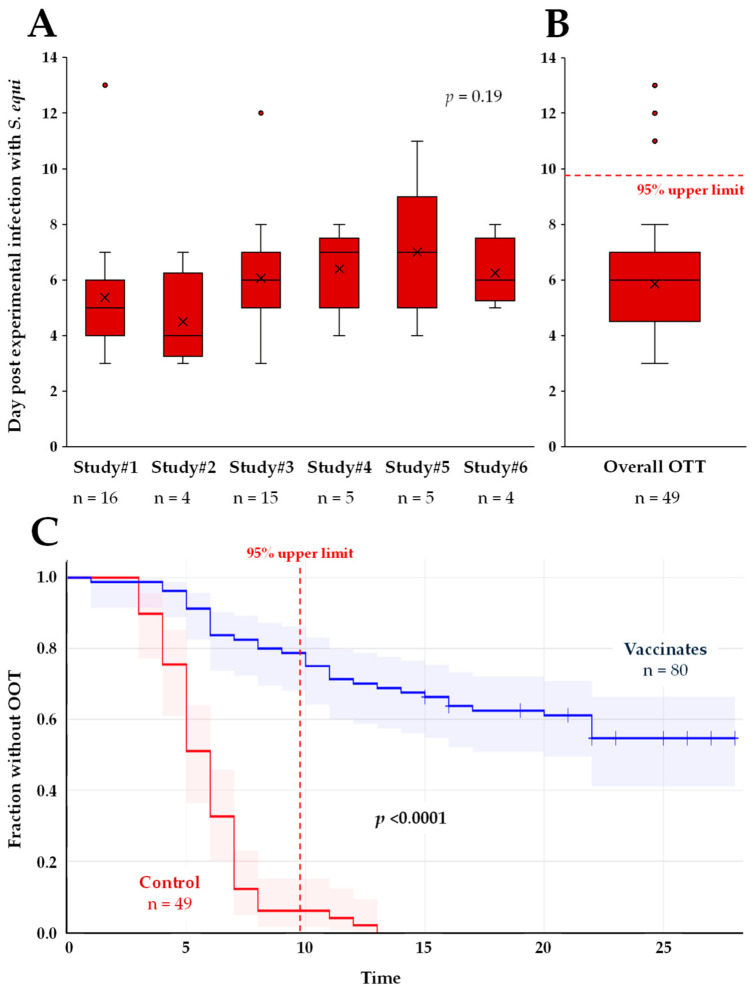
Onset of abnormal temperature (OOT; ≥39.0 °C). (**A**) OOT in control ponies per individual studies (*n* = 6). The Kruskal–Wallis H test *p* value is indicated. (**B**) Overall OOT in control ponies (*n* = 49). Data are presented as box-and -whiskers plots. The central line within each box represents the median, and the box shows the interquartile range. Whiskers extend to the minimum and maximum values within 1.5 × IQR. The mean is indicated by an ×, and outliers are shown as red circles. (**C**) Kaplan–Meier plot showing the probability of remaining OOT free with 95% confidence interval (shaded area); controls are in red (*n* = 49) and vaccinated ponies are in blue (*n* = 80). The Kaplan–Meier analysis *p* value is indicated. Significant *p* values (<0.05) are in bold text. The 95% upper limit of distribution is represented as a red dotted line.

The OOT was used as a marker of disease. Individual pony OOTs are reported in Supplementary Table S1. All 49 controls had a measurable OOT; 37 (46.25%) of the 80 vaccinated ponies reached the end of the observation period post-challenge without OOT (*p* < 0.0001). The Kruskal–Wallis H test indicated that there was no significant difference in OOT between the control groups across the individual studies (*n* = 49; χ^2^(5) = 7.43, *p* = 0.191; Figure 2A). The OOT for control ponies was 5.85 ± 2.12 days post-infection (95% CI: 1.77–2.65, median = 6 days, IQR 5–7, Figure 2B). Vaccination was associated with a significantly large delayed or absent OOT when compared with controls (Figure 2C). The log-rank test indicated a significant difference between control and vaccinated ponies (*p* < 0.0001). Based on the overall OOT distribution of the controls, a threshold of 10 days post-experimental infection was established based on the 95% upper limit of the control OOT distribution of 9.8 days post-infection. This threshold was used to delineate a significant delay in the OOT after experimental infection with *S. equi*. In total, 46 of the 49 controls (93.9%, 95% CI: 0.87–1) had an OOT < 10 days, compared with 17 (21.3%, 95% CI: 0.12–0.3) of the 80 vaccinated ponies, which indicates a significant large effect of the vaccination status on the OOT (2 × 2 Fisher exact test, *p* < 0.0001; Cramer’s V effect of 0.71). A similar result was obtained when the absence of abnormal temperature (37 vaccinated ponies and 0 controls) was defined as one of the outcome categories in the analysis (2 × 3 Fisher exact test, *p* < 0.0001; Cramer’s V effect of 0.71). Overall, the data set was considered to be balanced for subsequent ROC curve analyses, with 54 below the OOT threshold and 66 above the threshold.

### 3.2. IdeE, Eq85 and CCE Antibody Titres at the Time of Experimental Infection with S. equi

**Figure 3:**
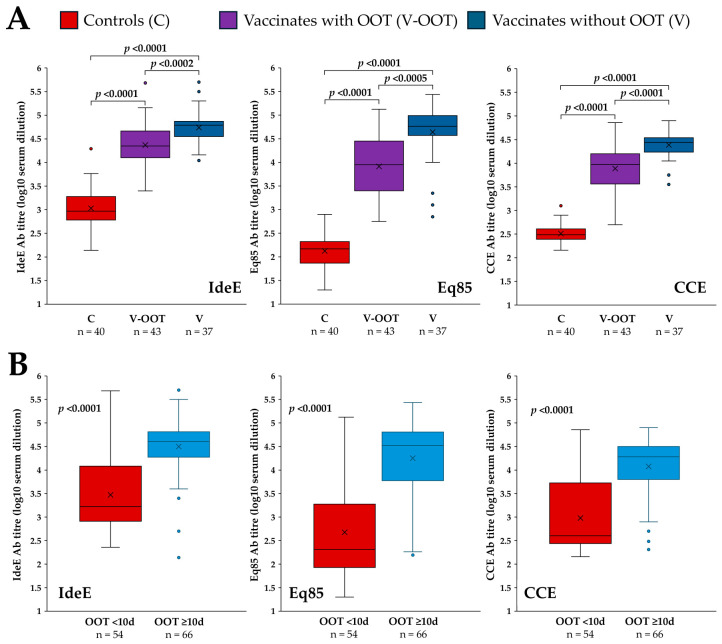
(**A**) IdeE, Eq85 and CCE Ab titres in control ponies (C; *n* = 40), vaccinated ponies with a measurable OOT or reaching a humane endpoint (V-OOT; *n* = 43) and vaccinated ponies that did not develop abnormal temperature by the end of study observation period (V; *n* = 37). The one-way ANOVA test (IdeE and CCE) and Mann–Whitney U test (Eq85) *p* values are indicated. (**B**) IdeE, Eq85 and CCE Ab titres in ponies with an OOT < 10 days (*n* = 54; red) or ≥10 days (*n* = 66; blue). The Mann–Whitney U test *p* value is indicated. Significant *p* values (<0.05) are in bold text. Data are presented as box-and -whiskers plots. The central line within each box represents the median, and the box shows the interquartile range. Whiskers extend to the minimum and maxi-mum values within 1.5 × IQR. The mean is indicated by an ×, and outliers are shown as red circles.

At the time of challenge, irrespective of the time since last vaccination, Ab titres to IdeE, Eq85 and CCE were significantly higher in vaccinated animals when compared with controls (*p* < 0.0001 for each Ag; IdeE 4.54 ± 0.43 vs. 3.03 ± 0.41; Eq85 4.25 ± 0.70 vs. 2.13 ± 0.40 and CCE 4.12 ± 0.48 vs. 2.51 ± 0.18, vaccinated vs. control ponies, respectively). All Ab titres were also significantly higher (*p* < 0.0005 for each Ag; Figure 3A) in vaccinated ponies that reached the end of the study observation period without developing an abnormal temperature (*n* = 37) when compared with other vaccinated animals (*n* = 43). The Cox analyses demonstrated a strong and highly significant association between IdeE, Eq85 and CCE Ab titres and the outcome (OOT and reaching end of study = right-censored). The regression coefficient for IdeE was −1.1145, corresponding to a hazard ratio of 0.3281 (95% CI: 0.25–0.43, *p* < 0.0001). The regression coefficient for Eq85 was −0.9785, corresponding to a hazard ratio of 0.3759 (95% CI: 0.3–0.48, *p* < 0.0001). The regression coefficient for CCE was −1.2874, corresponding to a hazard ratio of 0.276 (95% CI: 0.20–0.38, *p* < 0.0001). This indicates that higher IdeE, Eq85 or CCE Ab titres are strongly linked to longer time-to-event (OOT or reaching end of study; i.e., higher Ab titres make the event occur later). All associated global tests (likelihood ratio, Wald, and log-rank) were overwhelmingly significant (*p* < 0.0001), further confirming the robustness of this association. In all cases, the AFT models supported these findings, with IdeE, Eq85 and CCE Ab titres significantly increasing the event time (acceleration factor 2.14, 1.77 and 2.12, respectively; *p* < 0.0001).

When the OOT thresholds (i.e., ≥10 days) were used to categorise ponies (irrespective of the vaccination status), IdeE, Eq85 and CCE Ab titres were also significantly lower in ponies with an OOT < 10 days (*n* = 54) when compared with all other ponies (OOT ≥ 10 days and ponies that did not develop abnormal temperature; *n* = 66; *p* < 0.0001 for each Ag; IdeE 3.47 ± 0.75 vs. 4.5 ± 0.6; Eq85 2.67 ± 1 vs. 4.25 ± 0.77; CCE 2.98 ± 0.74 vs. 4.08 ± 0.60, OOT < 10 days vs. ≥10 days, respectively; Figure 3B).

### 3.3. Threshold of Protection

**Figure 4:**
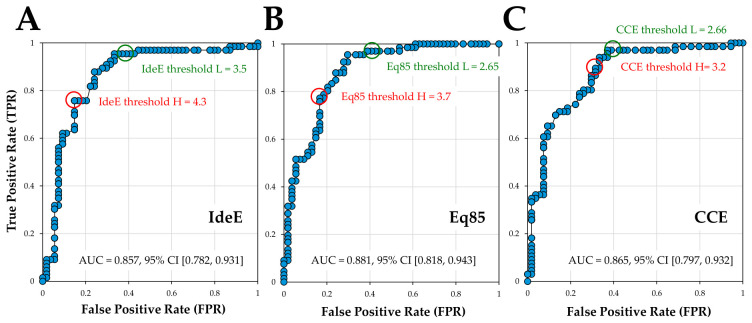
Receiver Operating Characteristic (ROC) curve analysis for IdeE (**A**), Eq85 (**B**) and CCE (**C**). The AUC and 95% CI are reported. The location of the high- and low-Ab titre thresholds are circled in red (Threshold-High) and green (Threshold-Low).

The threshold of 10 days post-experimental infection was used to define the outcome categories for subsequent ROC curve analyses (<10 days = 0, unprotected; ≥10 days and including animals that never developed abnormal temperature = 1, significantly delayed OOT-protected). ROC curve analyses showed that the Ab response to each Ag significantly discriminated between the protected and unprotected outcomes (IdeE area under the curve (AUC) = 0.86; Eq85 AUC = 0.88; CCE AUC = 0.86; *p* < 0.001 for each Ag; Figure 4). Consistent results were obtained using both analytical ROC testing and permutation-based inference (Monte Carlo *p* < 2.2 × 10^−16^ for each Ag).

Using the confusion matrix associated with each ROC curve analysis, Ab titre thresholds (low and high, Figure 4) were identified for each Ag to delineate the range of Ab titres with an accuracy value (overall correctness of the prediction) equal to, or greater than, 80%. Threshold-Low is the lowest Ab titre reaching an accuracy of 80%. Threshold-High maximises the precision and minimises the number of false positives (i.e., wrongly predicted as protected), while maintaining accuracy ≥80%. The threshold range was 3.5–4.3 for IdeE, 2.65–3.7 for Eq85 and 2.66–3.2 for CCE (Threshold-Low and -High, respectively). Precision represents the threshold ability to correctly predict protection (outcome = 1). The highest precision value (86.2%) was obtained with an IdeE Ab titre threshold of 4.3, followed by an Eq85 Ab titre of 3.7 (precision = 85.0%). Using these optimal thresholds, the specificity (ability to correctly predict the absence of protection) was 85.2% and 83.3%, respectively. Results from the confusion matrices are presented in Table 3. The precision value (77.7%) was lower for CCE (Ab titre threshold = 3.2).

A sequential combination of the IdeE and Eq85 thresholds (i.e., Eq85 threshold of 3.7 to be considered only if IdeE < 4.3) marginally increased the overall accuracy to 82.5%, the sensitivity to 86.4% and the NPV to 82.4% but slightly reduced other predictive values, as shown in Table 4. The addition of CCE did not further improve predictive values as 79 out of 80 vaccinated animals had CCE titres above the threshold of 3.2.

### 3.4. Duration of Immunity (DOI), Thresholds and Predicted Protection in Vaccinated Animals

**Figure 5:**
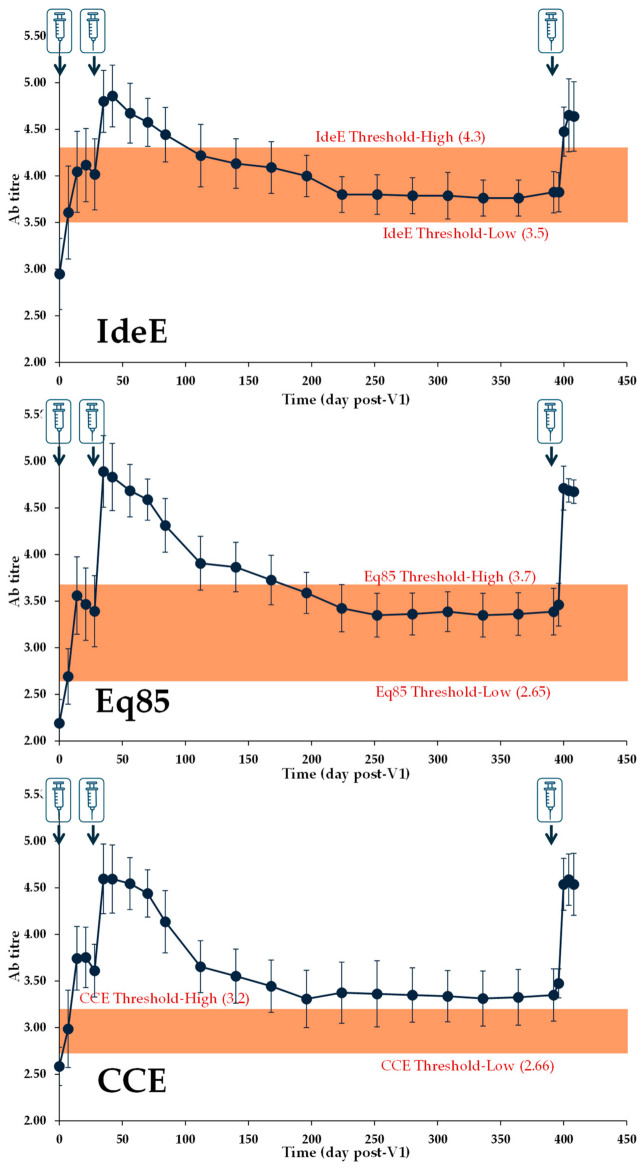
Duration of immunity: Kinetics of the Ab response to IdeE, Eq85 and CCE with V1 on D0, V2 on D28 and V3 on D392 (indicated by a syringe symbol). Antibody titres were measured up to 16 days post-V3; data are from Studies #1–#3 and Study #7 (EXP I in [15]). For each Ag, the Threshold-Low and -High boundaries defined in Table 3 (predicted with an accuracy ≥ 80%) are reported for reference (shaded area).

The DOI to each Ag, up to 12 months after V2, including the Ab response after boost immunisation (V3), is shown in Figure 5 (DOI; Studies #1–#3 and Study #7, EXP I in [15]). For all Ags, the lowest Ab titre measured after vaccination was above the respective Threshold-Low reported in Table 3.

Using the ROC curves and confusion matrices, the predictive values were calculated for the Ab titres measured 91, 168, 182 and 364 days after V2. Data are shown in Table 5. Overall, the accuracy remained above 79% for IdeE and Eq85 at all DOI time points. The precision for IdeE and Eq85 Ab titres measured 364 days after V2 were 78.2% and 81.1%, respectively. The number and frequency of protected vaccinated animals from Studies #1–#6 were calculated at the corresponding DOI titres and are presented in Table 5. With IdeE, Eq85 and CCE Ab titres equivalent to or greater than the ones measured 364 days after V2, 78.9% (60/76), 81.2% (56/69) and 77% (57/74) vaccinated ponies from Studies #1–#6 were protected, respectively (outcome = 1).

**Figure 6:**
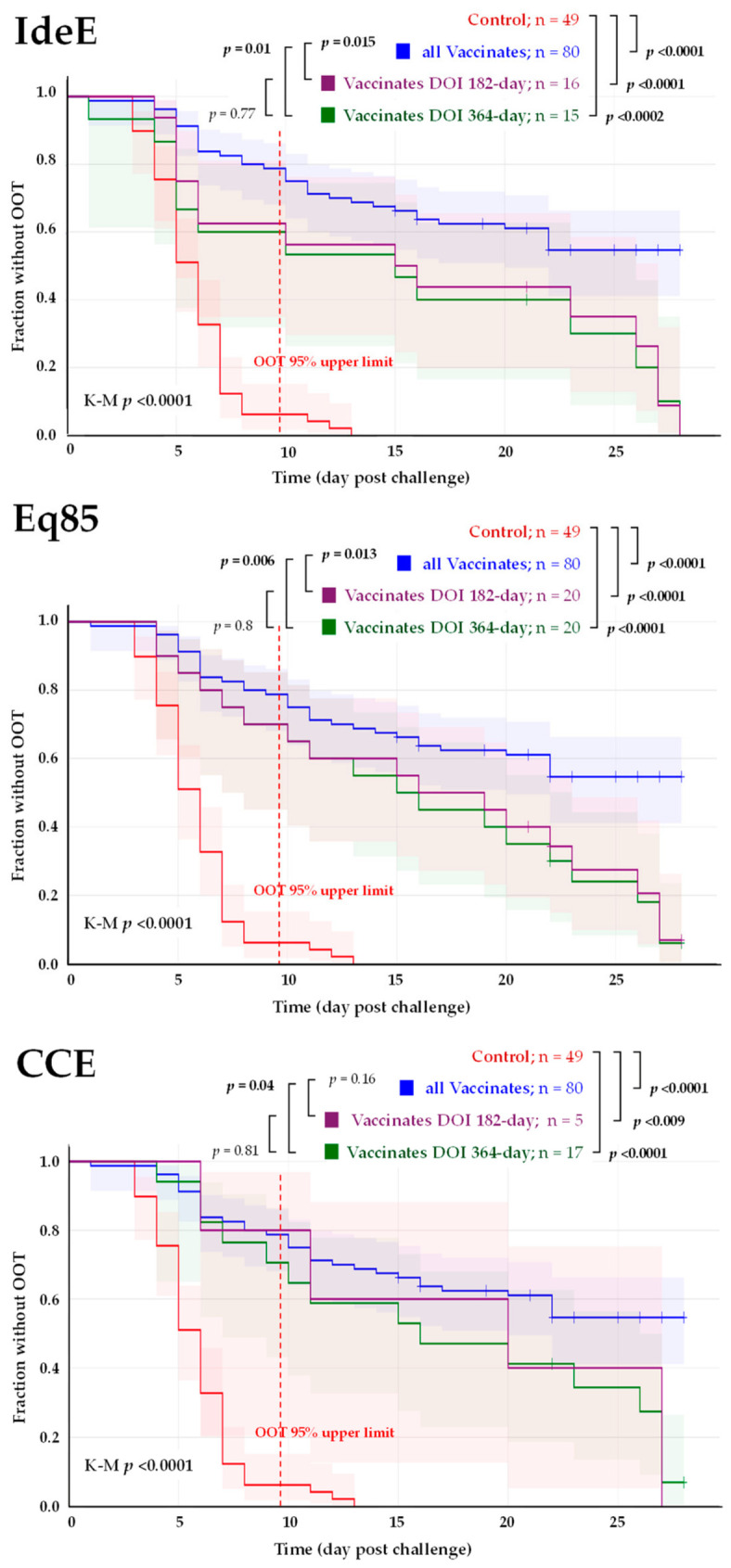
Duration of immunity: The Kaplan–Meier plot showing the probability of remaining OOT free with 95% confidence intervals (shaded area) is presented for each Ag. Controls are in red, all vaccinated ponies are in blue, vaccinated ponies with Ab titres in the 182-day subgroup are in purple and vaccinated ponies in the 364-day subgroup are in green. The Kaplan–Meier analysis (K-M) and the pairwise log-rank test *p* values are indicated for all 3 Ags. Significant *p* values (<0.05) are in bold text. n is the number of ponies in each group. The 95% upper limit of OOT distribution in controls is represented as a red dotted line.

Considering the Ab titres measured at 182 and 364 days after V2, the OOT in vaccinated ponies from Studies #1–#6 was compared with control ponies using Kaplan–Meier analysis (Figure 6). Vaccinated ponies were included in the DOI subgroups (182 or 364-day subgroups) if their Ab titres at challenge fell within the 95% distribution interval around the median titre measured at 182 or 364 days of immunity in Study #7. For IdeE, across all analyses, vaccination significantly delayed OOT compared with controls (all vaccinated animals, 182-day subgroup, and 364-day subgroup; *p* < 0.001). Ponies classified in the 182-day and 364-day titre-matched subgroups showed earlier OOT than the overall vaccinated population (*p* = 0.015 and *p* = 0.010, respectively); however, these two titre-matched subgroups did not differ significantly from each other. Similar results were obtained for Eq85. For CCE, only five vaccinated ponies fell within the 182-day range. Vaccination (all groups) significantly delayed OOT when compared with controls, but only ponies in the 364-day subgroup showed earlier OOT than the all-vaccinated-animals group (*p* = 0.04).

In total, 46 of the 49 controls (93.9%, 95% CI: 0.87–1) had an OOT < 10 days compared with 6 (40.0%, 95% CI: 0.15–0.65) of 15, 6 (30.0%, 95% CI: 0.1–0.5) of 20 and 5 (29.4%, 95% CI: 0.08–0.51) of 17 in the IdeE, Eq85 and CCE 364-day subgroups, respectively. At these Ab titre ranges (i.e., measured in Study #7 364 days after V2), significant relatively large to large effects of the vaccination status on the OOT were measured (2 × 2 Fisher exact test, *p* < 0.0001; Cramer’s V effect of 0.58 to 0.67).

## 4. Discussion

This retrospective study enabled the identification of IdeE and Eq85 Ab titres as correlates of protection against pyrexia, a key marker of clinical disease induced by experimental *S. equi* infection. In this study, elevated rectal body temperature was selected as a primary disease marker of choice for several reasons: (1) This measurement is inherently objective, unlike many other clinical signs that require operator assessment and scoring. (2) Pyrexia is the earliest clinical sign observed following experimental infection with *S. equi* [5,15]. (3) Pyrexia has been shown to correlate significantly with post mortem lesion scores and a composite clinical score of strangles (*p* < 0.001) [15]. (4) Pyrexia is a robust parameter, which developed in all unvaccinated control ponies in the retrospective studies. (5) The OOT was homogeneous across the six control groups and clearly distinguished controls from vaccinated animals. (6) The measurement of body temperature is routinely performed in the field to identify horses with early clinical signs of strangles, providing the possibility of conducting future field studies. The presence or absence of abscesses in the submandibular and retropharyngeal LNs at the time of euthanasia was also considered as a marker of disease. However, this parameter proved less reliable because unprotected vaccinated reached a study endpoint significantly later than controls, which provided additional time for *S. equi* to form abscesses in vaccinated, when compared with controls ponies that more quickly reached the welfare endpoint. Furthermore, the identification of abscesses, particularly those that develop in the retropharyngeal lymph nodes, is more difficult in the field. A number of vaccinated ponies (*n* = 37) reached the end of the study observation period (3 to 4 weeks, depending on the study) without developing OOT or meeting the predefined humane endpoint criteria. These animals were significantly associated with high IdeE, Eq85 and CCE Ab titres, supporting the role of these responses as CoP against *S. equi* infection. Thirty-two of these vaccinated ponies (86.5%; 32/37, 95% CI: 0.75–0.98) had no LN abscess or empyema upon post mortem examination.

Correlates of protection are invaluable for defining the expected levels of protection in horses vaccinated with Strangvac^®^ and to determine the duration of immunity. Although all three Ags provided good accuracy in the model, IdeE and Eq85 appeared to be a stronger CoP than CCE. Previous reports support the identification of Ab responses to IdeE as a mechanistic CoP (mCoP, a CoP mechanistically and causally responsible for protection [26]). The immunisation of seven Welsh mountain ponies with three doses of a prototype formulation of Strangvac, Septavacc, which comprised the recombinant proteins CNE, SclC, EAG, Eq5, Eq8, IdeE and IdeE2, protected six of seven ponies (86%) from developing fever after experimental infection with *S. equi* [42]. In comparison, administration of a vaccine containing five antigens (Pentavacc, lacking IdeE and IdeE2) resulted in the protection of only one of seven ponies (14%) [42]. Even though IdeE2 was shown to cleave equine IgG more effectively than IdeE, and thus may be more important to the virulence of *S. equi* [22], subsequent work demonstrated that adding IdeE2 to generate a nine-component vaccine did not enhance protection relative to the eight-component vaccine that contained IdeE but lacked IdeE2 [38]. These findings suggest that eliciting an immune response against IdeE, and thereby neutralising its associated virulence activity [23], is likely a key contributor to the protective effect observed following vaccination with Strangvac^®^. Although both Eq85 and CCE Ab titres were also significantly associated with protection and are regarded as CoP, their underlying functional activities remain poorly defined. Additional investigations are therefore needed to more fully characterise their functional roles and confirm their status as mCop.

The CoPs defined in this study apply to vaccination with Strangvac^®^ followed by experimental challenge using a high dose of *S. equi*. As discussed by Plotkin (2010), exposure to a large challenge dose may overwhelm vaccine-induced immunity [43]. In the clinical trials included in this retrospective analysis, each pony was experimentally infected with a dose ranging from 1.05 × 10^8^ to 2.3 × 10^8^ cfu, which represents a 10^4^- to 10^5^-fold increase over the minimum infectious dose required to infect immunocompetent horses [5,44]. Consequently, the CoP titres required to achieve protection under field conditions are expected to be lower than those analysed here. This expectation is consistent with the reported effectiveness of this vaccine during natural outbreaks of strangles [16,18], in which Strangvac^®^ was administered to horses during active circulation and exposure to *S. equi*. In these outbreaks, protection and reduced transmission were observed despite serological evidence of exposure of the vaccinated horses to *S. equi* before Ab titres had reached their peak (i.e., at 2 weeks after the second vaccination).

The model used in this retrospective study indicates that the Ab titres measured up to one year after the second vaccination [15] remain above CoP thresholds associated with significant levels of protection. Vaccinated animals with Ab titres equivalent to those measured 364 days after V2 were also significantly protected when challenged with *S. equi*. Furthermore, the functional activity of the IdeE-specific neutralisation response was demonstrated one year after V2 [23]. A serology-based predicted protection of 12 months after V2 is further supported by the concept of immunological memory, which is induced by vaccination in the context of a disease with a long incubation period, in which the memory response is boosted by exposure to the pathogen and can become effective prior to the development of clinical disease [43]. As a result, protection may still occur even when the Ab titre immediately prior to exposure falls below the calculated protection threshold. Following natural exposure to *S. equi*, the first clinical signs typically appear after approximately 14 days [5]. This window provides sufficient time for the infection to trigger a rapid anamnestic response from vaccine-induced memory cells, thereby contributing to effective protection.

The CoP described in this study will help to further characterise the protection induced by Strangvac^®^ in the field. They will also need to be validated in naturally infected horses, which present a humoral immune-response directed to dominant *S. equi* proteins that are not contained in Strangvac^®^, such as SeM and ScpC [45,46,47].

Although the precision calculated in this study is high (up to 82.5%), other immunological mechanisms induced by vaccination and/or infection, such as cell-mediated immunity, are likely to contribute to protection alongside the vaccine Ag-specific humoral response. The retrospective analyses conducted here involved six different clinical trials that utilised an experimental challenge that were conducted over a period of 5 years, including 49 unvaccinated control ponies (40 had serology suitable for CoP analysis) and 80 vaccinated animals. Although the data set included several independent experimental infection studies conducted over multiple years, the OOT in control ponies was highly consistent across these studies. The data set is considered balanced and sufficient for ROC curve analysis, allowing the detection of moderate to large AUC effects. There are some limitations in terms of precision and threshold optimisation as a single misclassification changes sensitivity or specificity by 1.5 to 1.8%. However, the re-use and integration of existing animal data align with the imperative to maximise the knowledge gained from animals already used in experimental studies, reducing the requirement for additional animal experimentation and adhering to the 3Rs principles [48]. The definition of CoP for Strangvac^®^ also provides new insights regarding the efficacy of this vaccine while minimising the need for future experimental challenges [49]. Finally, the protective thresholds calculated in this study were based on the Ab response to individual vaccine Ags. Bridging of these thresholds to the combined vaccine Ag iELISA and/or the single dilution iELISA [18] is warranted as these methods are more suitable for diagnostic settings.

## 5. Conclusions

Strangvac^®^ induces an immune response that is demonstrably associated with protection against *S. equi* infection. The Ab titres to the vaccine Ags IdeE, Eq85 and CCE constitute correlates of protection against clinical disease following experimental challenge, which will facilitate the identification of protection levels and the evaluation of herd immunity in horse populations in the field.

## Figures and Tables

**Table 1:** Nature and function of the antigens that are contained in Strangvac^®^. Ref. = References. SSp = sortase-processed surface protein.

Vaccine Ags	*S. equi* Protein	*S. equi* Localisation	Locus Tag in *Se*4047	Function	Ref.
IdeE	IdeE	secreted	SEQ0999	Endopeptidase, IgG cleavage, immune evasion	[22]
Eq85	Eq5	SSp	SEQ0256	Virulence factor, host interaction	[24]
	Eq8	SSp	SEQ0402		
CCE	CNE	SSp	SEQ0935	Collagen-binding protein	[19]
	SclC	SSp	SEQ2101	Collagen-like protein	[25]
	SclF	SSp	SEQ0855	Collagen-like protein	[25]
	SclI	SSp	SEQ1817	Collagen-like protein	[25]
	EAG	SSp	SEQ0721	α2-macroglobulin, IgG and albumin binding protein, immune evasion	[20]

**Table 2:** Details of the clinical trials included in this retrospective study. E. infection = experimental infection with *S. equi Se*4047 strain; Exp. = Experiment; ID = Identification; n/a = not applicable; Ref. = References if the study has been previously published.

Study ID ([Ref.])	Control Animals	Vaccinated Animals Day of Vaccination	E. Infection
Study #1 (Exp. II in [15])	*n* = 16 (placebo + 326 µg MatrixC adjuvant)	*n* = 16 (V1 + V2) D0 and D28 50 µg each Ag +326 µg MatrixC adj. i.m route	D42 (V2 + 2 weeks) Dose: 2 × 10^8^ cfu/pony
Study #2 (Exp. III in [15])	*n* = 4 (placebo + 326 µg MatrixC adjuvant) i.m route	*n* = 12 (V1 + V2) D0 & D28 50 µg each Ag +326 µg MatrixC adj. i.m route	D91 (V2 + 9 weeks) Dose: 1.7 × 10^8^ cfu/pony
Study #3 (Exp. IV in [15])	*n* = 15 (placebo + 326 µg MatrixC adjuvant) i.m route	*n* = 16 (V1–V3) D0, D28 and D119 50 µg each Ag + 326 µg MatrixC adj. i.m route	D133 V3 + 2 weeks Dose: 2.1 × 10^8^ cfu/pony
Study #4	*n* = 5 (placebo) i.m route (Exp. III/[38])	*n* = 18 3 groups (V1–V2) D1 and D43 30 µg to 300 µg each Ag + 326 µg MatrixC adj. i.m route	D57 (V2 + 2 weeks) Dose: 1.05 × 10^8^ cfu/pony
Study #5	*n* = 5 (placebo + 326 µg MatrixC adjuvant) i.m route	*n* = 8 (V1–V5) D1, D43, D127, D309, D400 50 µg each Ag + 326 µg MatrixC adj. i.m route	D414 (V5 + 2 weeks) Dose: 1.63 × 10^8^ cfu/pony
Study #6	*n* = 4 (placebo + MatrixC adjuvant) i.m route	*n* = 10 (V1–V3) D1, D43, D134 30 µg each Ag + 326 µg MatrixC adj. i.m route	D225 (V3 + 3 months) Dose: 2.3 × 10^8^ cfu/pony
Total number of ponies	*n* = 49	*n* = 80	
Study #7 (Exp. I in [15])	n/a	*n* = 12 (4/group; V1–V3) D1, D28, D119 (Group #1) or 210 (Group #2) or 392 (Group #3) 50 µg each Ag + 320–326 µg MatrixC adj. i.m route	n/a

**Table 3:** Confusion matrices and predictive values for IdeE, Eq85 and CCE. Low and high thresholds are reported for each Ag. Threshold value, Ab titre. P, Prediction; A, Actual. NPV, Negative Predictive Value. FN, false negative; FP, false positive; TN, true negative; TP, true positive. Values > 80% are in bold text. The 95% CI is reported in brackets.

**IdeE (Threshold-Low = 3.5)**			
	P. Protected	P. Unprotected	
A. Protected	TP: 63	FN: 3	**Sensitivity: 95.45%** [0.87–0.98]
A. Unprotected	FP: 21	TN: 33	Specificity: 61.1% [0.48–0.73]
	Precision: 75.0% [0.65–0.83]	**NPV: 91.7%** [0.78–0.97]	Accuracy *: 80% [0.72–0.86]
**IdeE (Threshold-High = 4.3)**			
	P. Protected	P. Unprotected	
A. Protected	TP: 50	FN: 16	Sensitivity: 75.8% [0.64–0.84]
A. Unprotected	FP : 8	TN : 46	**Specificity: 85.2%** [0.73–0.92]
	**Precision: 86.2%** [0.75–0.93]	NPV: 74.2% [0.62–0.83]	Accuracy *: 80% [0.72–0.96]
**Eq85 (Threshold-Low = 2.65)**			
	P. Protected	P. Unprotected	
A. Protected	TP: 64	FN: 2	**Sensitivity: 97.0%** [0.9–0.99]
A. Unprotected	FP: 22	TN: 32	Specificity: 59.3% [0.46–0.71]
	Precision: 74.4% [0.64–0.82]	**NPV: 94.1%** [0.81–0.98]	Accuracy *: 80% [0.72–0.86]
**Eq85 (Threshold-High = 3.7)**			
	P. Protected	P. Unprotected	
A. Protected	TP: 51	FN: 15	Sensitivity: 77.3% [0.66–0.86]
A. Unprotected	FP: 9	TN: 45	**Specificity: 83.3%** [0.71–0.91]
	**Precision: 85.0%** [0.74–0.92]	NPV: 75.0% [0.63–0.84]	Accuracy *: 80% [0.72–0.86]
**CCE (Threshold-Low = 2.66)**			
	P. Protected	P. Unprotected	
A. Protected	TP: 64	2	**Sensitivity: 97.0%** [0.9–0.99]
A. Unprotected	FP: 21	33	Specificity: 61.1% [0.48–0.73]
	Precision: 75.3% [0.65–0.83]	**NPV: 94.3%** [0.81–0.98]	Accuracy *: 80.8% [0.73–0.87]
**CCE (Threshold-High = 3.2)**			
	P. Protected	P. Unprotected	
A. Protected	TP: 59	7	**Sensitivity: 89.4%** [0.8–0.95]
A. Unprotected	FP: 17	37	Specificity: 68.5% [0.55–0.79]
	Precision: 77.7% [0.67–0.86]	**NPV: 84.1%** [0.71–0.92]	Accuracy *: 80% [0.72–0.86]

* Accuracy = (TP + TN)/total.

**Table 4:** Confusion matrix predictive values for the sequential combination of IdeE and Eq85. T = Threshold value = Ab titre. P, Prediction; A, Actual. NPV, Negative Predictive Value. FN, false negative; FP, false positive; TN, true negative; TP, true positive. Values > 80% are in bold text. The 95% CI is reported in brackets.

IdeE (T-High = 4.3) Then Eq85 (T-High = 3.7; Only If IdeE <T)			
	P. Protected	P. Unprotected	
A. Protected	TP: 57	FN: 9	**Sensitivity: 86.4%** [0.78–0.95]
A. Unprotected	FP: 12	TN: 42	**Specificity:** 77.8% [0.67–0.89]
	**Precision: 82.6%** [0.74–0.92]	**NPV: 82.4%** [0.72–0.93]	**Accuracy: 82.5%** [0.76–0.89]

**Table 5:** DOI Ab titres, associated accuracy (%) and frequency of protected vaccinated ponies in the model. The upper and lower threshold boundaries presented in Section 3.3 are kept as references (Ref. H and Ref. L, respectively). The Ab titres measured at V2 + 16 days (T (V2 + 16d)) are reported for reference. The closest rounded-down threshold available from the confusion matrices was selected for the DOI time points (T. applied). The number of vaccinated ponies (out of 80 vaccinated animals) with Ab titres above each applied threshold that were protected (V. prot.), or unprotected (V. un-prot.), are indicated. Frequency of protection and 95% CI (% Prot.) in vaccinated ponies (Studies #1–#6) with Ab titres ≥ DOI Ab titres measured in Study #7; *n* is the number measured at the specified time point (number of ponies); nV is the number of vaccinated animals with an Ab titre below the corresponding threshold in the model (out of 80 vaccinated ponies); Prot. = Protected; and V. = Vaccinated.

Ag./n	T (V2 + 16d)	DOI (V2+)	T DOI ^1^	T. Applied	Accuracy	V. Prot.	V. Unprot.	% Prot.	nV
**IdeE**			Ref. High	4.3	80	50	8	86.2 [0.77–0.95]	22
4	4.94 ± 0.25	+91d	4.08 ± 0.13	4.05	81.7	58	13	81.7 [0.73–0.91]	9
8	4.82 ± 0.57	+168d	4.00 ± 0.22	4.00	80.8	59	15	79.7 [0.71–0.89]	6
4	4.97 ± 0.51	+182d	3.99 ± 0.11	3.95	81.7	60	15	80.0 [0.71–0.89]	5
4	4.66 ± 0.66	+364d	3.83 ± 0.22	3.77	81.2	60	16	78.9 [0.70–0.88]	4
			Ref. Low	3.5	80.0	62	17	78.5 [0.69, 0.88]	1
**Eq85**			Ref. High	3.7	80.0	51	9	85.0 [0.76–0.94]	20
4	4.45 ± 0.3	+91d	3.61 ± 0.18	3.6	79.2	51	10	83.6 [0.74–0.93]	19
8	4.78 ± 0.22	+168d	3.59 ± 0.22	3.55	80.0	52	10	83.9 [0.69–0.88]	18
4	4.84 ± 0.2	+182d	3.59 ± 0.21	3.55	80.0	52	10	83.9 [0.75–0.93]	18
4	4.71 ± 0.25	+364d	3.39 ± 0.25	3.35	80.8	56	13	81.2 [0.72–0.90]	11
			Ref. Low	2.65	80.0	63	17	78.8 [0.70–0.88]	0
**CCE**			Ref. High	3.2	80.0	59	17	78.5 [0.69–0.88]	4
4	4.35 ± 0.39	+91d	3.49 ± 0.13	3.4	79.2	57	16	78.1 [0.69–0.88]	7
8	4.22 ± 0.42	+168d	3.31 ± 0.31	3.3	79.2	58	17	77.3 [0.68–0.87]	5
4	4.15 ± 0.17	+182d	3.17 ± 0.1	3.1	79.2	60	17	77.9 [0.69–0.87]	3
4	4.29 ± 0.6	+364d	3.35 ± 0.28	3.35	78.3	57	17	77.0 [0.67–0.87]	6
			Ref. Low	2.66	80.8	63	17	78.8 [0.70–0.88]	0

^1^ All DOI Ab titres were significantly greater (*p* < 0.008) than Ab titres in controls (*n* = 40).

## Data Availability

The original contributions presented in this study are included in the article/Supplementary Materials. Further inquiries can be directed to the corresponding author.

## Supplementary Materials

The following supporting information can be downloaded at https://www.mdpi.com/article/10.3390/vaccines14060533/s1, Table S1: Clinical examination scoring; Table S2: Rectal body temperature and antibody response; File S1: Details of unpublished clinical trials; File S2: Animal welfare information; Figure S1: Animal/data inclusion/exclusion.

## Abbreviations

The following abbreviations are used in this manuscript:

Ag	Antigen
Ab	Antibody
AUC	Area under the curve
CI	Confidence Interval
CoP	Correlate of Protection
DOI	Duration of Immunity
EAV	Equine artritis virus
EI	Equine influenza
EIV	Equine influenza virus
IQR	Interquartile range
LN	Lymph node
OOT	Onset of abnormal temperature
ROC	Receiver Operating Characteristic
*S. equi*	*Streptococcus equi* subspecies *equi*
SRH	Single Radial Haemolysis
VN	Virus-neutralising
