# Supplementary material for: Antibody Titres to Strangvac® Antigens Correlate with Protection and Duration of Immunity Against Experimental Infection with Streptococcus equi Subspecies equi
Source: Vaccines (Basel). 2026 Jun 16;14(6):533. doi: 10.3390/vaccines14060533 (PMC13307598; doi:10.3390/vaccines14060533)
Supplement: Supplementary file 1 [file vaccines-14-00533-s001.zip › Paillot et al Correlat Supp Table S2 Data.pdf]

Title: Antibody titres to Strangvac® antigens correlate with protection and duration of immunity against experimental infection with Streptococcus equi subspecies equi.

Supplementary Table S2: Individual Rectal Temperature (OOT) & Antibody response at the time of challenge. Status: C = placebo, V = vaccinates. ns = not sampled.

Pony	Study	Status	OOT	IdeE	Eq85	CCE
#1	Study#1	C	5	3.50	2.30	2.54
#2	Study#1	C	13	3.77	2.19	2.48
#3	Study#1	C	5	3.01	1.82	2.40
#4	Study#1	C	5	3.10	1.87	2.36
#5	Study#1	C	3	3.17	2.32	2.78
#6	Study#1	C	6	3.29	2.53	2.59
#7	Study#1	C	5	2.84	2.30	2.41
#8	Study#1	C	5	2.38	2.22	2.34
#9	Study#1	C	5	2.80	2.08	2.61
#10	Study#1	C	7	3.58	2.16	2.45
#11	Study#1	C	6	3.08	2.33	2.64
#12	Study#1	C	3	3.11	1.84	2.41
#13	Study#1	C	4	2.94	1.93	2.35
#14	Study#1	C	3	2.78	2.21	2.40
#15	Study#1	C	7	2.91	2.77	2.49
#16	Study#1	C	4	3.48	1.85	2.63
#17	Study#2	C	4	2.69	1.99	2.66
#18	Study#2	C	7	2.6	1.92	2.65
#19	Study#2	C	3	2.88	1.65	2.47
#20	Study#2	C	4	3.19	2.18	2.54
#21	Study#3	C	7	3.51	2.24	2.47
#22	Study#3	C	4	2.91	2.24	2.49
#23	Study#3	C	3	4.29	2.33	2.61
#24	Study#3	C	6	2.93	1.87	2.51
#25	Study#3	C	7	3.44	2.09	2.39
#26	Study#3	C	5	3.25	1.88	2.35
#27	Study#3	C	6	2.36	2.05	2.60
#28	Study#3	C	6	2.66	1.30	2.56
#29	Study#3	C	5	3.39	1.30	2.28
#30	Study#3	C	7	2.67	1.30	2.34
#31	Study#3	C	5	3.12	1.60	2.16
#32	Study#3	C	5	2.91	1.85	2.45
#33	Study#3	C	5	2.8	1.95	2.3
#34	Study#3	C	12	2.14	2.26	2.31

#35	Study#3	C	8	2.94	2.78	2.76
#36	Study#4	C	7	ns	ns	ns
#37	Study#4	C	7	ns	ns	ns
#38	Study#4	C	6	ns	ns	ns
#39	Study#4	C	4	ns	ns	ns
#40	Study#4	C	8	ns	ns	ns
#41	Study#5	C	7	3.4	2.8	3.1
#42	Study#5	C	11	2.7	2.85	2.9
#43	Study#5	C	4	3.15	2.65	2.6
#44	Study#5	C	7	2.6	2.9	2.5
#45	Study#5	C	6	3	2.4	2.65
#46	Study#6	C	6	ns	ns	ns
#47	Study#6	C	5	ns	ns	ns
#48	Study#6	C	6	ns	ns	ns
#49	Study#6	C	8	ns	ns	ns
#50	Study#1	V	9	4.97	4.28	3.56
#51	Study#1	V	21	4.54	4.60	4.45
#52	Study#1	V	21	4.84	4.90	4.57
#53	Study#1	V	8	5.68	4.74	4.14
#54	Study#1	V	10	4.66	4.39	3.93
#55	Study#1	V	6	4.48	5.12	4.86
#56	Study#1	V	16	4.71	4.62	4.50
#57	Study#1	V	11	4.70	4.29	4.45
#58	Study#1	V	21	4.84	4.80	4.37
#59	Study#1	V	11	5.16	4.28	4.30
#60	Study#1	V	21	4.59	5.14	4.90
#61	Study#1	V	21	5.50	5.36	4.44
#62	Study#1	V	5	4.56	4.50	3.98
#63	Study#1	V	10	4.39	4.28	3.61
#64	Study#1	V	14	4.84	4.52	4.50
#65	Study#1	V	16	4.67	4.43	4.72
#66	Study#2	V	21	4.61	4	4.39
#67	Study#2	V	1	4.22	4.61	4.43
#68	Study#2	V	21	4.56	4.1070	4.31
#69	Study#2	V	21	4.58	4.5732	4.47
#70	Study#2	V	5	4.18	4.5035	4.07
#71	Study#2	V	5	4.04	4.2225	4.00
#72	Study#2	V	21	4.04	4.87	4.34
#73	Study#2	V	21	4.57	4.63	4.18
#74	Study#2	V	19	4.16	4.255	4.29
#75	Study#2	V	6	4.28	4.45	3.97
#76	Study#2	V	21	4.33	4.25	4.33

#77	Study#2	V	12	4.61	4.52	4.42
#78	Study#3	V	21	4.66	5.15	4.63
#79	Study#3	V	21	4.87	4.87	4.90
#80	Study#3	V	6	4.78	4.40	4.41
#81	Study#3	V	21	4.92	4.76	4.27
#82	Study#3	V	21	4.87	4.95	4.51
#83	Study#3	V	21	4.53	5.03	4.12
#84	Study#3	V	21	5.07	5.43	4.71
#85	Study#3	V	21	4.79	4.57	4.29
#86	Study#3	V	21	4.83	4.88	4.68
#87	Study#3	V	21	4.81	4.84	4.51
#88	Study#3	V	21	4.92	4.75	4.80
#89	Study#3	V	21	5.2	4.69	4.48
#90	Study#3	V	21	4.8	4.62	4.33
#91	Study#3	V	21	4.87	5.09	4.20
#92	Study#3	V	21	4.74	5.05	4.59
#93	Study#3	V	21	4.93	4.76	4.45
#94	Study#4	V	4	3.75	3.6	3.9
#95	Study#4	V	28	4.35	4	4.5
#96	Study#4	V	10	4.2	3.3	2.75
#97	Study#4	V	23	4.1	3.45	3.4
#98	Study#4	V	6	3.85	2.8	3.9
#99	Study#4	V	28	4.3	3.1	3.75
#100	Study#4	V	15	4.2	3.95	3.8
#101	Study#4	V	27	4.1	3.7	3.55
#102	Study#4	V	22	4.35	3.4	4
#103	Study#4	V	26	4.2	3.55	3.8
#104	Study#4	V	27	3.95	2.75	3
#105	Study#4	V	13	3.65	3.15	2.7
#106	Study#4	V	28	4.2	4.05	4.1
#107	Study#4	V	27	4.6	3.8	3.2
#108	Study#4	V	16	4.15	3.8	3.5
#109	Study#4	V	11	3.4	3.8	3.3
#110	Study#4	V	20	3.6	3.3	3
#111	Study#4	V	26	4.75	4.6	3.9
#112	Study#5	V	25	5.7	4.75	4.5
#113	Study#5	V	25	4.8	4.95	4.05
#114	Study#5	V	25	4.75	4.65	4.15
#115	Study#5	V	25	4.8	5.15	4.5
#116	Study#5	V	22	4.55	4.7	4.2
#117	Study#5	V	25	4.7	4.7	4.05
#118	Study#5	V	25	5.3	5.25	4.7
#119	Study#5	V	17	4.65	4.45	4.15

#120	Study#6	V	22	4.3	2.85	3.55
#121	Study#6	V	6	4	3.2	3.6
#122	Study#6	V	19	4.35	3.5	4.2
#123	Study#6	V	7	4.95	3.25	3.7
#124	Study#6	V	5	4.2	3.5	4.05
#125	Study#6	V	22	4.4	3.35	4.05
#126	Study#6	V	15	4.3	3.1	4.1
#127	Study#6	V	6	4.4	2.75	3.35
#128	Study#6	V	8	4.4	3.4	4.4
#129	Study#6	V	4	4.05	3.35	3.8
