# Supplementary material for: Antibody Titres to Strangvac® Antigens Correlate with Protection and Duration of Immunity Against Experimental Infection with Streptococcus equi Subspecies equi
Source: Vaccines (Basel). 2026 Jun 16;14(6):533. doi: 10.3390/vaccines14060533 (PMC13307598; doi:10.3390/vaccines14060533)
Supplement: Supplementary file 1 [file vaccines-14-00533-s001.zip › Paillot et al Correlat Supp Table S1 scoring.pdf]

Title: Antibody titres to Strangvac® antigens correlate with protection and duration of immunity against experimental infection with Streptococcus equi subspecies equi.

Supplementary Table S1 : Clinical examination scoring.

<table>
<tr><th colspan="2">Area</th><th>Observation</th><th>Score</th></tr>
<tr><td colspan="2">Temperature</td><td>°C</td><td>Not applicable</td></tr>
<tr><td colspan="2">Ocular</td><td>0=normal, 1=serous, 2=mucopurulent, bl=bilateral</td><td>0, 1, 2 or double</td></tr>
<tr><td colspan="2">Nasal</td><td>0=normal, 1=serous, 2=mucopurulent, bl=bilateral</td><td>0, 1, 2 or double</td></tr>
<tr><td colspan="2">Lymph node</td><td>0=normal, 1=slight, 2=moderate, 3=severe,
4=abscessate, 5=sinus and drain, bl=bilateral</td><td>0, 1, 2, 3, 4, 5 or double</td></tr>
<tr><td colspan="2">Cough</td><td>0=not present, 1=present, 2=marked</td><td>0, 1 or 2</td></tr>
<tr><td colspan="2">Swallow</td><td>0=normal, 1=painful</td><td>0 or 1</td></tr>
<tr><td colspan="2">Feeding</td><td>0=normal, 1=reluctant, 2=off feed</td><td>0, 1 or 2</td></tr>
<tr><td colspan="2">Demeanour</td><td>0=normal, 1=depressed, 2=markedly depressed</td><td>0, 1 or 2</td></tr>
<tr><td rowspan="3">Injection site reaction</td><td>Heat</td><td>0=normal, 1=slight, 2=moderate, 3=severe</td><td>0, 1, 2 or 3</td></tr>
<tr><td>Pain</td><td>0=normal, 1=slight, 2=moderate, 3=severe</td><td>0, 1, 2 or 3</td></tr>
<tr><td>Swelling*</td><td>0=normal, 1=slight, 2=moderate, 3=severe
*If present measure width and length</td><td>0, 1, 2 or 3</td></tr>
</table>
