# Supplementary material for: Antibody Titres to Strangvac® Antigens Correlate with Protection and Duration of Immunity Against Experimental Infection with Streptococcus equi Subspecies equi
Source: Vaccines (Basel). 2026 Jun 16;14(6):533. doi: 10.3390/vaccines14060533 (PMC13307598; doi:10.3390/vaccines14060533)
Supplement: Supplementary file 1 [file vaccines-14-00533-s001.zip › Paillot et al Correlate Supp Figure S1.pdf]

Title: Antibody titres to Strangvac® antigens correlate with protection and duration of immunity against experimental infection with Streptococcus equi subspecies equi.

Supplementary Figure S1: Animal/data inclusion/exclusion

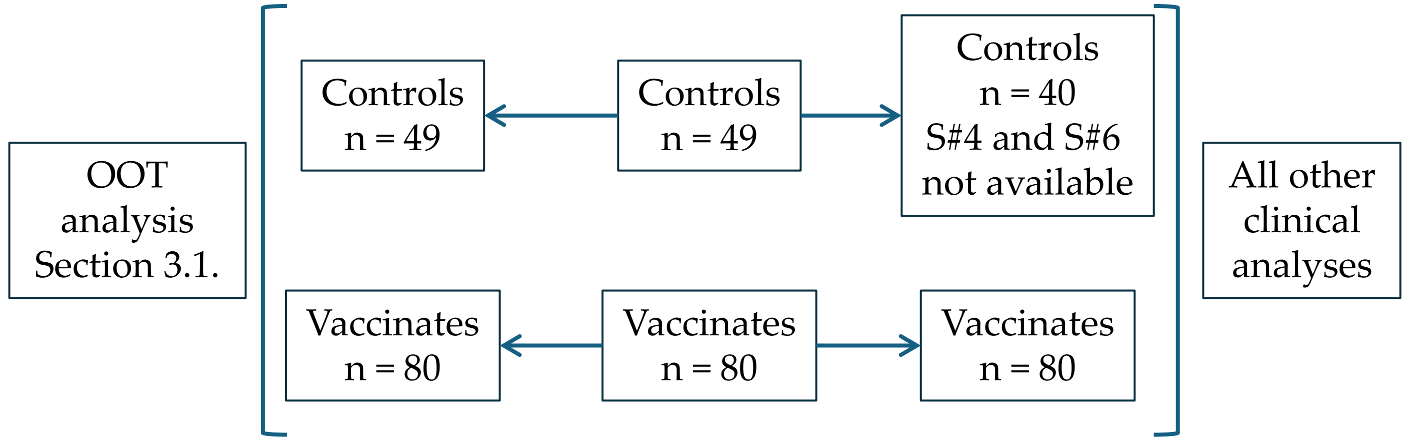

Number of animals per study per time point				
**Time point**	**Study #1**	**Study #2**	**Study #3**	**Study #7**
**-1/0**	16	12	16	12
**7**	16	12	16	
**14**	16	12	16	
**21**	16		16	
**28**	16		16	8
**35/36**	16	12	16	8*
**41/42**	16*	12	16	
**56**			16	4
**70**			16	
**84**			16	12
**112**				12
**140**				8
**168**				8
**196**				8
**224**				4
**252**				4
**280**				4
**308**				4
**336**				4
**364**				4
**392**				4
**396**				4
**400**				4
**404**				4
**408**				4

* D41 for Study #1; D36 for Study #7

DOI section 3.4.
