# Supplementary material for: Antibody Titres to Strangvac® Antigens Correlate with Protection and Duration of Immunity Against Experimental Infection with Streptococcus equi Subspecies equi
Source: Vaccines (Basel). 2026 Jun 16;14(6):533. doi: 10.3390/vaccines14060533 (PMC13307598; doi:10.3390/vaccines14060533)
Supplement: Supplementary file 1 [file vaccines-14-00533-s001.zip › Paillot et al Correlate Supp File S2 Animal W PROOF.pdf]

Title: Antibody titres to Strangvac® antigens correlate with protection and duration of immunity against experimental infection with Streptococcus equi subspecies equi.

Supplementary File S2: Animal welfare information

Further information:

Further details of the serology responses post-vaccination and the protection conferred against Experimental challenge for Studies #1-#3 and #7 are provided in Robinson et al., 2020 [15].

Ethical considerations:

All studies were conducted under the auspices of a Home Office Project License according to the Animal Scientific Procedures Act 1986 and following ethical review and approval by the Animal Health Trust's Animal Welfare and Ethical Review Body (RPP 01_08, approved May 2008; reviewed and approved December 2012).

Animal housing and management:

All ponies were housed on the Animal Health Trust premises prior to, and during the Experiments according to AHT/SOP/EQU/04 - Routine Husbandry Procedures for Ponies.

All ponies were kept at pasture on grass or in suitable barns with nearby facilities for sampling and vaccination. Drinking water was available *ad libitum* and nutrition was provided in an amount necessary to keep the ponies in good condition. Regular veterinary health checks with certification were conducted.

Studies #1 to #6 were randomised, double-blinded and placebo controlled studies on the efficacy of vaccination against *S. equi* challenge. Following the vaccination period, ponies were moved to the Allen Centre for Veterinary Studies (a purpose-built containment facility at the Animal Health Trust) prior to challenge. Ponies were separated randomly into up to four different rooms (depending of the number of ponies in the study). A maximum of eight ponies per room (6.4 m^2 per pony) was adhered to. Drinking water was provided *ad libitum* and nutrition was provided as necessary to keep the ponies in good condition. Each animal room was enriched by the use of long fibre feed (Haylage), mineral licks, molasses and flavoured licks, play balls, feed balls, radio and maintaining contact with members of their original peer group. The temperature of each room was maintained in accordance with Home Office Recommendations.

Health and welfare monitoring protocols:

During the vaccination phase of these studies, clinical observations for the occurrence of local and/or systemic reactions were performed prior to and after vaccination according to SOP/EQU/08 (Pony Clinical Observations).

All ponies were examined daily for three days pre-vaccination, for 14 days post each vaccination and, in Studies #1 to #6, for three days pre-challenge. Animals showing signs of ill health were examined at the earliest opportunity to establish a diagnosis.

Following challenge and for up to 21-28 days (depending of the study), each pony was examined once daily in the morning for the occurrence of clinical signs associated with *S. equi* infection and scored according to Robinson et al., 2020 [15]. A second visual examination was also performed in the afternoon for the purposes of animal welfare.

Animal welfare endpoints:

In Studies #1 to #6, the humane end-point of pyrexia and a preference for haylage and water over dried pelleted food was used post-challenge. Ponies were euthanased on reaching the humane end-point and prior to the onset of more severe clinical signs or complications associated with *S. equi* infection.

Fate of experimental animals:

All 12 ponies in Study #7 completed the study and were released to the Animal Health Trust's pony herd.

All of the ponies in Studies #1 to #6 completed the vaccination phase of these studies and went on to be challenged with *S. equi* strain 4047 as described previously [15]. In these studies, all placebo ponies were euthanased on reaching the humane end-point. Forty-three vaccinates were euthanased on reaching the humane end-point. Thirty-seven vaccinates were euthanased on reaching the end of the studies at 21 to 28 days post-challenge, depending of the study [15].

Reference:

15. Robinson, C.; Waller, A.S.; Frykberg, L.; Flock, M.; Zachrisson, O.; Guss, B.; Flock, J.-I. Intramuscular Vaccination with Strangvac Is Safe and Induces Protection against Equine Strangles Caused by Streptococcus Equi. *Vaccine* **2020**, *38*, 4861–4868, doi:10.1016/j.vaccine.2020.05.046.
