# Supplementary material for: Antibody Titres to Strangvac® Antigens Correlate with Protection and Duration of Immunity Against Experimental Infection with Streptococcus equi Subspecies equi
Source: Vaccines (Basel). 2026 Jun 16;14(6):533. doi: 10.3390/vaccines14060533 (PMC13307598; doi:10.3390/vaccines14060533)
Supplement: Supplementary file 1 [file vaccines-14-00533-s001.zip › Paillot et al Correlate Supp File S1 Study out PROOF.pdf]

Title: Antibody titres to Strangvac® antigens correlate with protection and duration of immunity against experimental infection with Streptococcus equi subspecies equi.

Supplementary File S1: Studies #5 and #6 outlines

Study #5 outline

This study was designed to determine the efficacy of the Strangvac vaccine administered via the intramuscular route using a five-dose regimen with challenge occurring 2 weeks post final vaccination (**Table S3**). Fourteen Welsh mountain ponies (approximately 6 months of age at the time of first vaccination) were enrolled in the study. The occurrence of adverse reaction to vaccination was recorded up to two weeks after each immunisation. Serum samples were obtained up to six months post third and three months post forth vaccination to determine if vaccination had induced an immunological memory. Challenge two weeks post fifth vaccination was performed to mimic the effects of vaccination in the face of an outbreak to determine if this strategy could be effective for the prevention of strangles. One vaccinated pony was withdrawn from the study for unrelated reasons. All 13 remaining ponies were challenged with an identical dose of $1.63 x 10^8$ cfu of S. equi strain 4047, which was split and administered via both nostrils. Clinical signs of diseases were measured up to 25 days post-challenge.

Table S3: Study #5 design

Group	Vaccination	Challenge
Vaccinated<br>n = 9	D1, D43, D127, D309, D400<br>50µg each Ags + 326µg MatrixC adj.<br>i.m route	D414<br>n = 8
Control<br>n = 5	placebo+326 µg MatrixC adjuvant)<br>i.m route	D414<br>n = 5

Study #6 outline

This study was designed to determine the efficacy of the Strangvac vaccine administered via the intramuscular route using a three-dose regimen with challenge occurring 3 months post final vaccination (**Table S4**). Fourteen Welsh mountain ponies (approximately 6 months of age at the time of first vaccination) were enrolled in the study. The occurrence of adverse reaction to vaccination was recorded up to two weeks after each immunisation. Serum samples were collected every months after the first immunisation to confirm seroconvertion. Challenge was performed three months post third vaccination to determine confirm a three months duration of immunity. All 14 ponies were challenged with an identical dose of $2.33 x 10^8$ cfu of S. equi strain 4047, which was split and administered via both nostrils. Clinical signs of diseases were measured up to 22 days post-challenge.

Table S4: Study #6 design

Group	Vaccination	Challenge
Vaccinated<br>n = 10	D1, D43, D134<br>30µg each Ag + 326 µg MatrixC adj.<br>i.m route	D225<br>n = 10
Control<br>n = 4	Placebo + MatrixC adjuvant)<br>i.m route	D225<br>n = 4
